# Exome Sequencing Diagnoses X-Linked Moesin-Associated Immunodeficiency in a Primary Immunodeficiency Case

**DOI:** 10.3389/fimmu.2018.00420

**Published:** 2018-03-05

**Authors:** Gabrielle Bradshaw, Robbie R. Lualhati, Cassie L. Albury, Neven Maksemous, Deidre Roos-Araujo, Robert A. Smith, Miles C. Benton, David A. Eccles, Rod A. Lea, Heidi G. Sutherland, Larisa M. Haupt, Lyn R. Griffiths

**Affiliations:** ^1^Genomics Research Centre, School of Biomedical Sciences, Institute of Health and Biomedical Innovation, Queensland University of Technology, Brisbane, QLD, Australia

**Keywords:** lymphopenia, whole exome sequencing, moesin, *MSN*, X-linked moesin-associated immunodeficiency

## Abstract

**Background:**

We investigated the molecular etiology of a young male proband with confirmed immunodeficiency of unknown cause, presenting with recurrent bacterial and Varicella zoster viral infections in childhood and persistent lymphopenia into early adulthood.

**Aim:**

To identify causative functional genetic variants related to an undiagnosed primary immunodeficiency.

**Method:**

Whole genome microarray copy number variant (CNV) analysis was performed on the proband followed by whole exome sequencing (WES) and trio analysis of the proband and family members. A >4 kbp deletion identified by repeated CNV analysis of exome sequencing data along with three damaging missense single nucleotide variants were validated by Sanger sequencing in all family members. Confirmation of the causative role of the candidate gene was performed by qPCR and Western Blot analyses on the proband, family members and a healthy control.

**Results:**

CNV identified our previously reported interleukin 25 amplification in the proband; however, the variant was not validated to be a candidate gene for immunodeficiency. WES trio analysis, data filtering and *in silico* prediction identified a novel, damaging (SIFT: 0; Polyphen 1; Grantham score: 101) and disease-causing (MutationTaster) single base mutation in the X chromosome (c.511C > T p.Arg171Trp) *MSN* gene not identified in the UCSC Genome Browser database. The mutation was validated by Sanger sequencing, confirming the proband was hemizygous X-linked recessive (–/T) at this locus and inherited the affected T allele from his non-symptomatic carrier mother (C/T), with other family members (father, sister) confirmed to be wild type (C/C). Western Blot analysis demonstrated an absence of moesin protein in lymphocytes derived from the proband, compared with normal expression in lymphocytes derived from the healthy control, father and mother. qPCR identified significantly lower *MSN* mRNA transcript expression in the proband compared to an age- and sex-matched healthy control subject in whole blood (*p* = 0.02), and lymphocytes (*p* = 0.01). These results confirmed moesin deficiency in the proband, directly causative of his immunodeficient phenotype.

**Conclusion:**

These findings confirm X-linked moesin-associated immunodeficiency in a proband previously undiagnosed up to 24 years of age. This study also highlights the utility of WES for the diagnosis of rare or novel forms of primary immunodeficiency disease.

## Introduction

Primary immunodeficiency disorders (PIDs) encompass a diverse group of mostly inherited genetic disorders that compromise immune system function and predispose individuals to recurrent infections and other immune disorders such as autoimmunity, hematological disorders and lymphoid malignancies (1–3). As defined by the International Union of Immunological Societies, there are almost 300 single gene defects described which are causative for the wide range of phenotypes (4), making PIDs a rapidly expanding field of medicine. Loss of function mutations in multiple genes coding for proteins that regulate the actin cytoskeleton are known to cause PID, the most well characterized form being Wiskott-Aldrich (WAS) syndrome (5). This is a rare X chromosome-linked PID where expression and function of the WAS protein results in failure of Arp2/3-mediated actin polymerization causing a combined defect of innate and adaptive immunity associated with microthrombocytopenia, eczema, blood cancers, and increased risk of autoimmunity (5).

Next-generation DNA sequencing technologies such as whole genome sequencing (WGS) and whole exome sequencing (WES) have revolutionized the field of genomics by enabling a high-throughput and increasingly cost-effective method for complex and rare disease diagnosis (6). While WGS is the most comprehensive technique able to identify around 4 million variants within the entire genome, including coding and non-coding regions, WES provides more focus on only protein-coding exons, which harbor around 85% of pathogenic mutations (7, 8). Currently, WES permits lower operating costs (US$800 per sample compared to US$1,500 per sample for WGS) with faster data generation and less complex analysis, however, it is unable to identify structural variants (6, 9). Here, we used WES to elucidate possible disease-causing variants in a case diagnosed previously as immunodeficient of unknown cause.

## Background

The proband is a 24-year–old male. At age 7 weeks, he had boils in his groin and axillae. At 18 months of age, he had a prolonged episode of bronchitis for which he received antibiotics but was not hospitalized. He suffered repeated upper respiratory tract infections and ear infections for the next few years. He had two episodes of chicken pox (Varicella) at around 18 months and 3 years. The first episode was severe, with widespread skin lesions, but no secondary infection or hemorrhagic lesions. At about 3 years of age, he had a persistent eye infection, which progressed to periorbital cellulitis, secondary to *Pseudomonas aeruginosa* infection. Neutropenia was first noted during that episode. Bone marrow aspirate showed normal myeloid maturation, consistent with an immune etiology. No treatment was started at that stage. At 4 years of age, he had an episode of thrombocytopenia, with platelets falling to <5. A further bone marrow aspirate was consistent with an immune mediated thrombocytopenia, and he responded rapidly to intravenous immunoglobulin. He had some further skin infections and paronychiae at this time, and continued to suffer repeated respiratory infections, although not requiring hospitalization. At 6 years 6 months of age, he had his first episode of pneumonia and had several hospitalizations.

At this stage, a diagnosis of combined immunodeficiency was made: he had persistent absolute lymphopenia, normal T-cell receptor Vβ distribution, low numbers of T-cell receptor excision circles (TRECs), no mutation in the common γ chain gene, low IgG, IgA, and IgM. He was negative for HIV by PCR. He started immunoglobulin replacement (IVIg) and G-CSF treatment in early 2000 and has very much improved symptomatically since then, however, lymphocyte counts have remained severely decreased (Tables 1 and 2).

**Table 1:** Hematology laboratory workup for the proband.

Hematology	June 28, 2001	June 17, 2004	June 14, 2005	Units	Ref interval
Age at evaluation	8 years	11 years	12 years		6–12 years
Hemoglobin	118	127	127	g/L	110–15
Hematocrit	0.34	0.36	0.37		0.36–0.46
Red cell count	–	4.3	4.5	×10^12^/L	4.0–5.6
Mean cell volume	87	84	84	fL	77–95
White cell count	2.4 **L**	2.5 **L**	3.4 **L**	×10^9^/L	5.0–12.0
Neutrophils	2.04	2.00	2.67	×10^9^/L	1.5–7.5
Lymphocytes	0.32 **L**	0.40 **L**	0.52 **L**	×10^9^/L	1.0–6.5
Monocytes	0.04	0.10	0.11	×10^9^/L	0–1.5
Eosinophils	–	0.00	0.05	×10^9^/L	0–0.6
Basophils	–	0.00	0.01	×10^9^/L	0–0.20
Platelets	363	329	401	×10^9^/L	150–600
Lymphocyte subsets					
Lymphocytes	370 **L**	ND	ND	Cells/μL	1,900–3,700
T-cells CD3+	74 **L**	ND	ND	Cells/μL	600–2,600
T-helper CD4+	33 **L**	ND	ND	Cells/μL	650–1,500
T suppressor CD8+	33 **L**	ND	ND	Cells/μL	370–1,100
B-cells CD19+	15 **L**	ND	ND	Cells/μL	270–860
NK cells CD16+/56+	3 **L**	ND	ND	Cells/μL	4–20
TRECs	<100,000 **L**	ND	ND	Per million PBMC	100,000
Protein studies					
Immunoglobulin G (IgG) on IVIg	805	ND	ND	mg/dL	520–1,098
Immunoglobulin A (IgA)	16 **L**	ND	ND	mg/dL	36–230
Immunoglobulin M (IgM)	30 **L**	ND	ND	mg/dL	42–172

*Historical hematology workup showing persistent leucopenia with lymphopenia and hypogammaglobulinemia at 8, 11, and 12 years of age. Neutropenia improved with G-CSF treatment*.

*PBMC, peripheral blood mononuclear cells; IVIg, intravenous immunoglobulin; ND, not determined*.

*Red font indicates values lower than the reference interval*.

**Table 2:** Hematology laboratory workup for the proband.

Hematology	December 16, 2016	July 04, 2017	Units	Ref interval
Age at evaluation	23 years	24 years		>12 years
Hemoglobin	138	142	g/L	135–175
Hematocrit	0.42	0.41		0.40–0.54
Red cell count	4.8	4.9	×10^12^/L	4.5–6.5
Mean cell volume	87	84	fL	80–100
White cell count	1.5 **L**	1.3 **L**	×10^9^/L	3.5–10.0
Neutrophils	0.96 **L**	0.74 **L**	×10^9^/L	1.5–6.5
Lymphocytes	0.46 **L**	0.47 **L**	×10^9^/L	1.0–4.0
Monocytes	0.06	0.08	×10^9^/L	0–0.9
Eosinophils	0.00	0.00	×10^9^/L	0–0.6
Basophils	0.01	0.02	×10^9^/L	0–0.15
Platelets	316	255	×10^9^/L	150–400
Lymphocyte subsets	0.46 **L**	ND	×10^9^/L	1.0–4.0
T-cells CD3+	0.43 **L**	ND	×10^9^/L	0.75–2.50
T-helper CD4+	0.15 **L**	ND	×10^9^/L	0.50–1.90
T suppressor CD8+	0.24	ND	×10^9^/L	0.21–1.2
B-cells CD19+	0.01 **L**	ND	×10^9^/L	0.05–0.60
NK cells CD16+/56+	0.02 **L**	ND	×10^9^/L	0.05–0.60
Protein studies				
Immunoglobulin G (IgG) on IVIg	9.15	6.98	g/L	5.76–15.36
Immunoglobulin A (IgA)	0.49 **L**	0.45 **L**	g/L	1.24–4.16
Immunoglobulin M (IgM)	0.13 **L**	0.21 **L**	g/L	0.48–3.1

*Current hematology results showing persistent leucopenia with neutropenia, lymphopenia, and hypogammaglobulinemia at 23 and 24 years of age. Peripheral blood lymphocyte surface markers shows all lymphocyte subsets are below the normal range excluding T suppressor CD8+ cells which are on the lower level of normal*.

*IVIg, intravenous immunoglobulin; ND, not determined*.

*Red font indicates values lower than the reference interval*.

We previously investigated a functional molecular cause for this undiagnosed immunodeficiency through whole genome arrays. Briefly, analysis of Affymetrix 250K SNP microarray data of the case and a matched healthy control subject *via* a copy number analysis tool identified hyperploidy of a region centromeric to chromosome 14q11.2, mapped over the interleukin 25 (*IL25*) gene (10). IL-25 (a member of the IL-17 family of cytokines) induction in mice has been previously associated with a T-helper (Th) 2-like pathological immune response (11) and shown to regulate the development of autoimmune inflammation mediated by IL-17-producing cells (12, 13) and was deemed a plausible candidate for further analyses.

Green et al. (10) paired genetic analysis with transcriptional profiling and found a large number of genes associated with Th1/Th2 profiles to be differentially expressed in peripheral blood lymphocytes of the proband, indicative of a Th2 bias confirmed by flow cytometric analysis. T-cells from the proband and control subject were cultured and activated *in vitro* with qPCR analysis demonstrating higher *IL25* expression in the proband when compared to the control. The skewed Th2 immunity hypothesized in the proband was in line with his susceptibility to infections normally cleared by Th1 responses, such as Varicella and *Pseudomonas*. Further *in vitro* studies were performed in B-cell line models to measure the effect of IL-25 treatment on cellular proliferation and viability, however when proband and control lymphocytes were treated with exogenous IL-25, no differences were observed (data not shown).

## Materials and Methods

### Whole Exome Sequencing

Peripheral blood samples were obtained from all participants and genomic DNA (gDNA) extracted using the QIAamp DNA Blood Maxi Kit (Qiagen) according to the manufacturer’s instructions. Exome sequencing of the family trio (case, mother, and father) was performed on the Ion Chef and Ion Proton Next Generation Sequencing platform using the Ion Ampliseq Exome RDY Kit 4 × 2 (Life Technologies). DNA was quantitated using the Agilent 2100 Bioanalyser using the Agilent High Sensitivity DNA kit (Agilent Technologies) with 50 ng gDNA used for library preparation of each family member. Barcode adapters were ligated to each exome library with the Ion Express Barcode Adapters 1-96 Kit (Life Technologies). Using a Qubit 2.0 Fluorometer (Life Technologies), libraries were diluted to a concentration of 100 pM before being clonally amplified on the Ion Chef System using the Ion PI Chip v3 (Life Technologies) prior to loading on an Ion PI Chip v3 for sequencing *via* semiconduction.

### WES Analysis

Raw sequences from each library were aligned to the GRCh37/Hg19 reference genome *via* the Ion Torrent Server TMAP alignment algorithm. Trio analysis was performed on the Ion Reporter Suite V.5.0 (Life Technologies) where variant annotation identified included single-nucleotide polymorphisms (SNPs) and indels (insertions and deletions) for each exome library. At a total read-depth of 20×, the target base coverage was 94.63%. A combined total of 51,997 variants were identified with 482 of these unique to the proband. Variant filtering then focused on deleterious variants (frameshift insertion and deletion, stoploss, missense, and nonsense) followed by screening using the OMIM public database as a filter for genes related to immunological disorders. 13 identified variants were then further filtered using *in silico* missense variant effect prediction tools with the following parameters designated as damaging: SIFT < 0.05, PolyPhen > 0.8, and Grantham Score > 100. SIFT and PolyPhen predict both the structural and functional impact of a variant based on properties such as accessible surface area, ligand contacts, solvent accessible area, and change in residue side change volume (14). The Grantham score focuses on the difference between amino acid atomic composition, polarity, and volume where a radical amino acid substitution gives a score above 100 (15). MutationTaster predicts whether a variant is tolerable, polymorphic, or damaging and disease-causing to identify potential disease-causing variants (16). Three final candidate variants were then verified using the Integrative Genome Viewer (IGV) software (Broad Institute). Copy number variant (CNV) analysis was performed on WES data for the case and family members using the same filtering steps as described. WES was used to indicate the general presence of the CNV, which was validated by touchdown PCR and Sanger sequencing. A more detailed description of the methods used can be found in the BMC Bioinformatics paper by Demidov et al. (17). With the ongoing advances in sequencing technology and improvement in alignment techniques for large-scale data analysis, we were able to confirm that *IL25* does not lie within the previously identified amplified region on chromosome 14, but rather 4 Mb downstream.

### Sanger Sequencing

PCR was performed using forward and reverse primers for the *MSN* (F: 5′-TTCTCTCCTGCACAGGGACTTT-3′; R: 5′-ATTCACCCTGTAAGGGAAGTGGG-3′), *TET2* (F:5′-GCCTGATGGAACAGGATAGAAC-3′;R:5′-TTCCCTTCATACAGGGTATTC-3′), and *NLRP8* (F:5′-ATATCCAGCGCCTGATAGCG-3′; R:5′-TCAGGGTGACGGTCAGTT-3′) single nucleotide variants (SNVs). Following amplification, PCR products were treated with Exo-SAP for use in Big Dye Terminator v3.1 sequencing reactions, analyzed on the 3500 Genetic Analyzer (ThermoFisher Scientific, Life Technologies). Sequencing data for each sample chromatogram was assessed using Chromas Lite 2.1.1 software.

### Lymphocyte Isolation, Activation, and Expansion *In Vitro*

Peripheral blood mononuclear cells (PBMCs) were isolated from heparinized whole blood from the family trios and a healthy control subject, using the Ficoll-Histopaque centrifugation method (Sigma-Aldrich). Isolated PBMCs were washed in 1X PBS and plated into T-75 culture flasks in RPMI-1640 culture medium (Invitrogen, ThermoFisher) with phytohemagglutinin (PHA) mitogen for up to 24 h to allow monocytes to adhere to the bottom of the flask. After 3 h lymphocytes in suspension were removed and plated into a new T-75 flask with PHA and IL-2 (Sigma-Aldrich). Lymphocytes were allowed to proliferate in RPMI-1640 culture medium at 37°C in 5% CO_2_ for up to 5 days, and if necessary further expanded after 2 days. Live lymphocyte counts were performed on a hemocytometer and viability assessed by Trypan blue dye. Cell lysates were prepared for qPCR and Western Blot analyses with 4 × 10^5^ to 3 × 10^6^ cells.

### Total RNA Extraction, cDNA Synthesis, and qPCR

Whole blood from a healthy control subject and family members was collected in PAXgene™ vacutainer tubes for immediate stabilization of intracellular RNA at collection. Total RNA was extracted using the PreAnalytiX PAXgene™ Blood RNA Kit according to the manufacturer’s instructions. Lymphocytes were harvested in TRIzol and total RNA extracted using the Direct-zol™ RNA MiniPrep kit (Zymo Research). Total RNA from whole blood and lymphocytes was converted to cDNA using the iScript™ cDNA Synthesis Kit (Bio-Rad) according to the manufacturer’s instructions. Samples for qPCR were assayed in triplicate using the Promega SYBR Green with 18S expression used as an endogenous control for normalization of the data. The ^ΔΔ^Ct comparison method was used to measure relative gene expression on the QuantStudio™ 7 instrument (ThermoFisher) and analyzed using Student’s *T*-test to calculate statistical significance (*p* < 0.05).

### Western Blotting

Total protein was extracted from cell lysates using Runx protein-lysis buffer (containing protease and phosphatase inhibitors) as previously described (18). Protein concentration was measured using the Qubit™ Protein Assay Kit (Invitrogen). A Western Blot using 30 µg protein per sample was performed using an anti-*MSN* primary antibody (ab52490, Abcam) and an HRP-conjugated secondary antibody (anti-Rabbit IgG #7074, Cell Signaling). Sample loading was normalized using HRP-conjugated anti-Beta-actin (#5125S, Cell Signaling). Detection of target protein was carried out with ECL (Clarity™ ECL, Bio-Rad) using the Fusion Spectra chemiluminescent system (Vilber Lourmat, Fisher Biotec) and optical density quantitation assessed using Bio-1D software.

### Ethics and Cell Line Validation

This study was performed in accordance with the recommendations of the Queensland University of Technology Human Research Ethics Committee (Approval number 1400000125). All subjects gave written informed consent in accordance with the Declaration of Helsinki. Written informed consent was also obtained from the proband and family members for the publication of this case report. Cell line validations were performed for commercial Toledo non-Hodgkin lymphoma (NHL) B-cell and MCF-7 breast cancer cell lines (data not shown).

## Results

### Identification and Validation of a SIRPβ1 Deletion

Copy number variant analysis of the WES data identified a large >4 kbp deletion in the *SIRPβ1* gene in the proband (chr20:1,576,140–1,580,375 Hg38) not present in other family members. Further inspection suggested the deletion in the proband encompassed exon 2 of the *SIRPβ1* gene. Validation of this deletion by touchdown PCR and Sanger sequencing confirmed a deletion of exon 2, which has high sequence homology with another exon in the same gene (data not shown). Further Sanger validation to determine the size of the deletion was discontinued due to high sequence homology in that region. Furthermore, this exon 2 deletion has also been found in a number of healthy individuals (19); and therefore, this variant was considered to be an unlikely pathogenic candidate for the immunodeficiency.

### Identification and Validation of a Hemizygous X-Linked MSN Mutation

**Figure 1:**
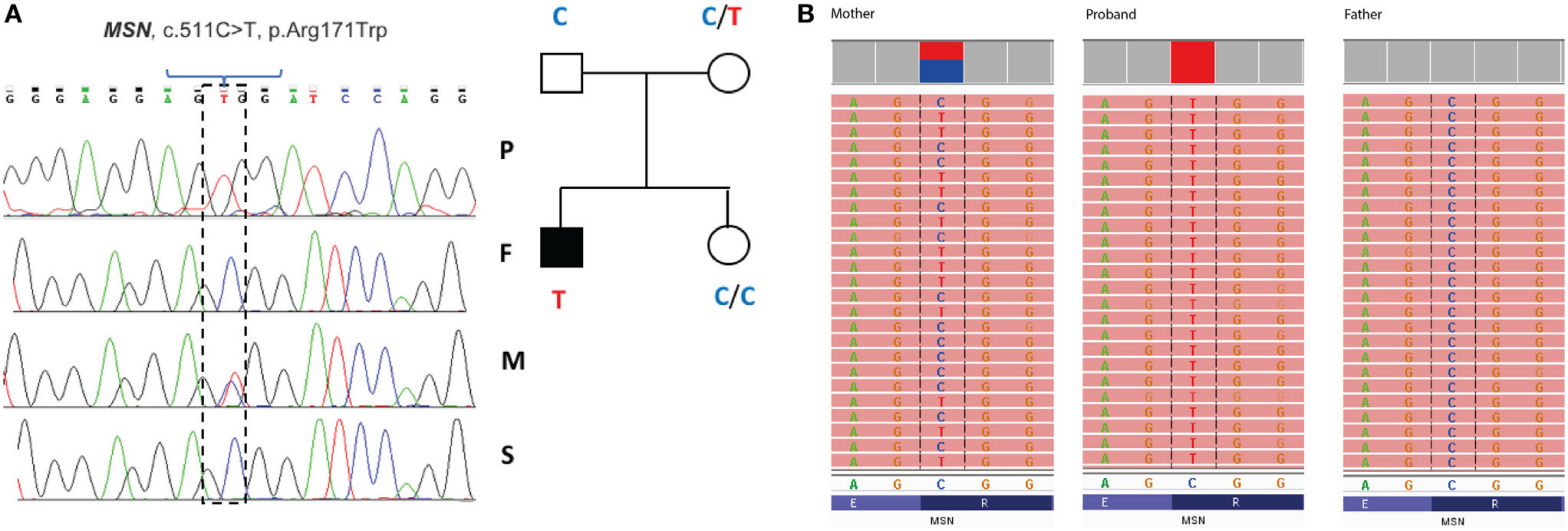
Validation of the ^R171W^MSN mutation in family members. **(A)** Sanger sequence and pedigree tree diagram for the missense *MSN* C > T mutation. P, proband; F, father; M, mother; S, sister. Mother is heterozygous C/T marked by a double blue and red peak and proband is hemizygous –/T marked by a single red peak. **(B)** IGV software showing *MSN* genotypes for the mother (C/T), proband (–T), and father (C/C), respectively. IGV, integrative genomics viewer.

Filtering of the WES data on the Ion Reporter Suite yielded three variants in three immune function genes *TET2, NLRP8* and *MSN* with damaging SIFT and Polyphen scores predicted to be disease-causing/polymorphic by MutationTaster (Table 3). The *TET2* SNP (rs111948941, C > T) has a frequency of 0.15. The homozygous case (T/T) inherited an affected allele from each parent, both of whom are heterozygous (C/T) at this locus. The *NLRP8* variant (rs754128390, A > C) is very rare, occurring in <1/10,000 individuals. The proband was identified to be *de novo* heterozygous (A/C) with both parents homozygous wild type (A/A) at this locus. The *MSN* variant (c.511C > T, p.Arg171Trp at position chrX:64951012, Hg19) was a novel mutation, not reported in the genomic databases or the literature at the time of query. The proband is hemizygous X-linked for the mutation (-/T), inheriting the affected allele from his heterozygous (C/T) mother. The *TET2* SNP and *NLRP8* rare variant occur in healthy individuals with no known disease associations, therefore, they were considered unlikely candidates and not analyzed further. WES coverage for the *MSN* mutation was 145× for the proband, 119× for the father, 150× for the mother, with an average total coverage of 138×. The *MSN* mutation was validated by Sanger sequencing (Figure 1A) and confirmed using IGV for whole genome and exome data in all four family members (Figure 1B) and as such, considered to be the most likely candidate.

**Table 3:** Whole exome trio analysis data filtering and *in silico* prediction tools (SIFT, PolyPhen, Grantham Score, and MutationTaster) identified three damaging and disease-causing SNVs in the proband: *MSN* C > T, *TET2* C > T, and *NLRP8* A > C.

Gene	Locus (Hg19)	dbSNP/transcripts	Nucleotide change	MAF	Proband genotype	Variant effect	Amino acid change	SIFT	PolyPhen	Grantham score	Mutation taster
*MSN*	ChrX:64951012	Novel mutationNM_002444	c.511C > T	n/a	T/–	Missense	p.Arg171Trp	0	1	101	Disease-causing
					Hemizygous X-linked	AA change	Basic to non-polar residue change	Damaging	Damaging	Radical amino acid change	

*TET2*	Chr4:106155199	rs111948941 NM_001127208 NM_017628	c.100C > T	T = 0.015 (dbSNP)	T/T	Missense	p.Leu34Phe	0	0.5	22	Disease-causing
					Homozygous Recessive	AA change	Both residues are non-polar	Damaging	Possibly damaging	Conservative AA change

*NLRP8*	Chr19:56467265	*De novo* variantNM_176811	c.1841A > C	C = < 1/10,000 (gnomAD)	A/C	Missense	p.His614Pro	0	> 0.8	77	Polymorphic, minor allele frequency; Hg19, GRCh37 reference genome.ly-morphic
					Heterozygous	AA change	Basic to non-polar residue change	Damaging	Damaging	Non-conservative AA change	

*MAF, minor allele frequency; Hg19, GRCh37 reference genome*.

*Red font highlights significant damaging and disease-causing prediction scores*.

### Protein Detection and Quantification

**Figure 2:**
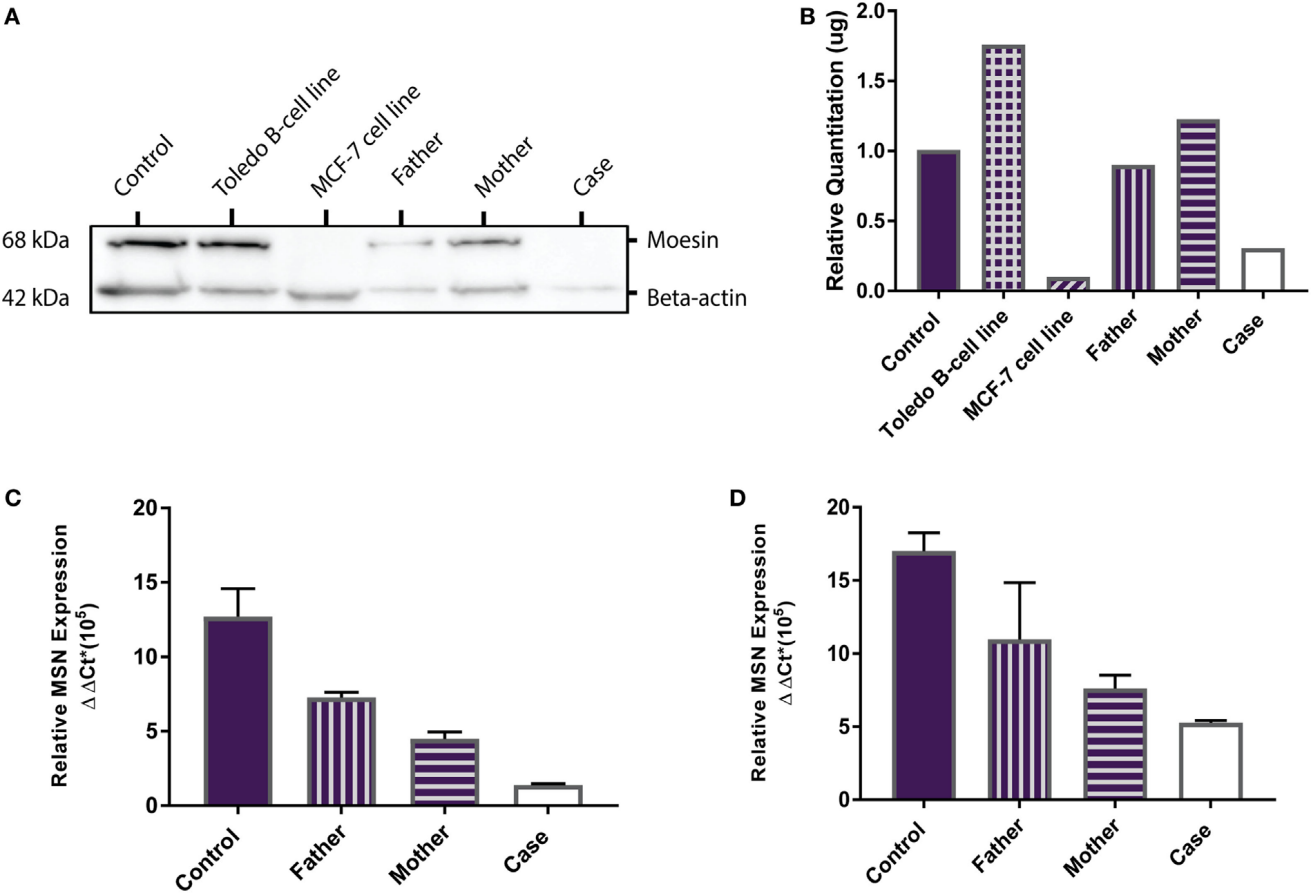
MSN protein detection and *MSN* gene expression. **(A)** Western Blot shows absence of the 68 kDa moesin protein in the MCF-7 cell line (negative control, lane 3) and in the proband (lane 6), and presence of moesin in the healthy control subject (lane 1), Toledo B-cell line (positive control, lane 2), father (lane 4), and mother (lane 5). Beta-actin (42 kDa) was used as the loading control. **(B)** Optical density protein quantitation software showed very low levels of moesin (<0.5 μg) in the MCF-7 cells and the proband, confirming a moesin deficiency. **(C)** qPCR analysis of *MSN* mRNA transcript levels in whole blood (PAXgene) from the control subject, father, mother and proband. Error bars depict standard error of the mean (SEM) between triplicates. **(D)** qPCR analysis of *MSN* mRNA transcript levels in lymphocytes from the control subject, father, mother, and case. Error bars depict SEM between triplicates.

*MSN* encodes the moesin protein (MSN) which is a member of the ezrin-radixin-moesin (ERM) family of cell structure-related proteins. Western Blot analysis confirmed the presence of the 68 kDa MSN protein in the Toledo NHL B-cell line (positive control) and absence of MSN in the MCF-7 breast cancer cell line (negative control). MSN was shown to be present in the healthy control, father, mother, and is absent in the proband (Figure 2A). Relative MSN concentrations were normalized against beta-actin concentrations where the MCF-7 cells and the proband show very low MSN concentrations (<0.5 μg; Figure 2B). These data confirm the proband has a MSN protein deficiency, not present in the healthy control or parents, and is the most likely sole genetic cause for the persistent lymphopenia.

### MSN mRNA Transcript Expression

*MSN* gene expression was measured in all samples (healthy control, father, mother and proband). In whole blood the proband had significantly lower moesin mRNA expression than the control (*p* = 0.02), father (*p* = 0.002) and mother (*p* = 0.01) and the mother had significantly lower expression than the father (*p* = 0.01) and control (*p* = 0.04) (Figure 2C). In isolated lymphocytes, the proband (*p* = 0.01) and mother (*p* = 0.004) had significantly lower expression than the control (Figure 2D). Overall expression levels in all participants were higher in lymphocytes than in whole blood, with *MSN* mainly expressed in lymphocytes, however, it is also expressed in monocytes, neutrophils and platelets. The lower levels observed in the mother compared to the father and control and higher levels than the proband are likely due to the heterozygous C > T mutation in the *MSN* gene. High levels were observed in the healthy control who was age- and sex-matched to the proband.

## Discussion

The variant identified here in the *MSN* gene is a missense, hemizygous, single-base mutation in the proband. This mutation was not identified in current online databases (UCSC Genome Browser, gnomAD) with no reference number and no disease association reported in OMIM at the time of query (March 2016). A genome-wide association study (GWAS) search in the GWAS Catalog (20) produced no results for disease-trait-associated variants in the *MSN* gene, or other polymorphisms within the *MSN* gene.

More recently an OMIM entry for Immunodeficiency-50 was identified describing a novel X-linked moesin-associated immunodeficiency (X-MAID) (21). This was the first study to document a moesin-associated disease in humans and describes six male cases from four unrelated families with the same ^R171W^MSN missense mutation as identified in the proband. A seventh case had a different mutation, p.R553X, which caused a frameshift and truncation of MSN in the F-actin binding domain. The authors show an impact on lymphocyte function *in vitro* where moesin deficiency impaired proliferation following activation with mitogen, increased adhesion, and reduced migration (21).

Moesin is a member of the ERM family of cell structure-related proteins that regulate the cell’s actin cytoskeleton (5, 22). The mutation identified results in a radical amino acid substitution from basic arginine to non-polar tryptophan within the functional four-point-one, ezrin, radixin, moesin domain. However, interestingly both our study and that of Lagresle-Peyrou et al. suggest that rather than interfering with protein function, the ^R171W^MSN mutation triggers degradation of moesin mRNA in lymphocytes with a loss of expression at the transcript and protein level (21). Moesin has been shown to have a crucial and non-redundant role in lymphocyte homeostasis in mice (23). As such, a lack of moesin protein could prevent efficient lymphocyte migration and egress from lymphoid organs causing persistent absolute lymphopenia in the peripheral blood, as observed in the case. The low TRECs exhibited by the proband at a young age, are an indicator of recent thymic emigrants (24). We compared the Lagresle-Peyrou et al. cases’ (P1-P7) and proband phenotypes in more detail and we surmise the P3 case would be the most similar to the proband in terms of clinical outcomes due to both individuals presenting with no eczema, no *Molluscum contagiosum*, and presentation of an autoimmune reaction in the form of TTP (P3) and ITP (proband). Considering we cannot confirm whether the P3 case has an *IL25* or *NLRP8* variant which could play a role in the absence of eczema or *M. contagiosum*, it would be difficult to determine whether these variants are playing a role in the proband. In addition, as there are no outstanding differences between the proband and all of the other cases, we can be more confident that the ^R171^MSN mutation is the sole molecular cause for this disease in the proband (Table 4).

**Table 4:** Comparison of clinical outcomes for cases P1–P7 (Lagresle-Peyrou et al.) and the proband.

Case	*MSN*mutation	Bacterial infections	Varicella zoster	Eczema	*Molluscum contagiosum*	Autoimmunity	Persistent lymphopenia	Fluctuating neutropenia	IgG therapy	Improvement with G-CSF	Alive and well
P1	R171W	Yes	Yes	Yes	Yes	No	Yes	Yes	Yes	No G-CSF	Yes
P2	R171W	Yes	Yes	Yes	Yes	No	Yes	Yes	Yes	Yes	Yes
**P3**	R171W	Yes	Yes	No	No	TTP	Yes	Yes	Yes	No GCS-F	Yes
P4	R171W	Yes	Yes	Yes	No	No	Yes	Yes	Yes	Yes	Yes
P5	R171W	Yes	No	Yes	No	No	Yes	Yes	Yes	No G-CSF	Yes
P6	R171W	Yes	Yes	Yes	Yes	No	Yes	Yes	Yes	No G-CSF	Yes
P7	R553*	Yes	No	No	No	No	Yes	Yes	Yes	No G-CSF	Yes
**Proband**	R171W	Yes	Yes	No	No	ITP	Yes	Yes	Yes	Yes	Yes

*Red font highlights similarities between case P3 and proband*.

Infants with genetic defects of the immune system that cause severe combined immunodeficiency, or SCID, are effectively identified by population-wide screening practices in the United States (25). Newborns are routinely screened for SCID by using qPCR to quantitate TRECs from DNA extracted from dried blood spots (24); however, this is a laborious assay with a number of limitations. Recently, a newborn case with an ^R171W^MSN mutation was identified by WES (26) when undergoing screening for SCID. Together with our report of an Australian case with X-MAID due to the ^R171W^MSN mutation, this data suggests that although rare, it is a site of recurrent mutation.

## Concluding Remarks

Together with our case report, the findings by Lagresle-Peyrou et al. and Delmonte et al. confirm the effectiveness and validity of WES as a diagnostic method for PIDs and SCID, as well as an investigative method for the identification of novel or rare variants causing novel forms of PID not yet been documented. Identification of these rare and novel variants early in the patient’s life will not only aid with swift intervention prior to life-threatening infections, but may also provide possibility for application of gene therapies and personalized treatment options for these patients.

## Ethics Statement

This study was performed in accordance with the recommendations of the Queensland University of Technology Human Research Ethics Committee (approval number 1400000125). All subjects gave written informed consent in accordance with the Declaration of Helsinki. Cell line validations were performed for commercial Pfeiffer non-Hodgkin lymphoma (NHL) B-cell and MCF-7 breast cancer cell lines (data not shown).

## Author Contributions

GB performed candidate gene validation, designed qPCR primers, performed cell culture, functional experiments and analysis, and wrote the first draft of the manuscript; RRL, NM performed exome sequencing trio analysis; RRL performed candidate gene validations and drafted the exome sequencing method and analysis sections of the manuscript; CA, NM, RAL, MB, DE performed sequencing data analysis and interpretation. DR-A, CA, NM, and RS designed PCR primers for candidate gene validations. HS, LH, and LG conceptualized the study, contributed to data interpretation and edited the final version of the manuscript. LG provided funding support and finalized the manuscript for submission.

## Conflict of Interest Statement

The authors declare that the research was conducted in the absence of any commercial or financial relationships that could be construed as a potential conflict of interest.
